# Trauma-Addiction Link Among Women in Harm Reduction

**DOI:** 10.1192/j.eurpsy.2025.934

**Published:** 2025-08-26

**Authors:** S. Matmati, E. Khelifa, A. Mtiraoui, C. Bey

**Affiliations:** 1 razi hospital, tunis; 2 psychiatry, hopital farhat hached, sousse, Tunisia

## Abstract

**Introduction:**

The life journeys of women who have experienced childhood violence are often marked by great complexity. This study aimed to better understand the links between childhood trauma and the development of substance use disorders, in order to propose therapeutic interventions tailored to the specific needs of these women

**Objectives:**

identify the most frequent types of trauma and their frequency in the studied sample. To describe the most commonly used substances, patterns of use, and the severity of substance use disorders.

**Methods:**

sample of 53 women who frequented harm reduction centers participated in this study. The Childhood Trauma Questionnaire (CTQ) was employed to evaluate experiences of childhood trauma, while the Drug Use Disorder Identification Test (DUDIT) was utilized to assess the severity of substance use disorders. Data collection was conducted anonymously, and subsequent analysis was performed using statistical software spss.

**Results:**

In our study, 53 women consulting risk reduction centers met the inclusion criteria.

- A history of childhood trauma (CTQ ≥ 35) was observed in 92.5% of cases (n=49). Severe trauma scores were found in:
- 45% for emotional neglect
- 23% for physical neglect
- 51% for emotional abuse
- 59% for physical abuse
- 21% for sexual abuse
- A drug dependence (DUDIT ≥ 2) was observed in 72% of cases (n=38), i.e., all women in the study who used psychoactive substances. The most frequent dependencies were related to cannabis (53%), followed by benzodiazepines (41%) and pregabalin (36%).
- Injectable drug use was observed in 23% of cases, with Subutex being the most common (n=10; 5%). Two women used heroin (1%).

**Conclusions:**

These results highlight the importance of integrating a trauma-informed approach into the care of women with substance use disorders. Specific therapies, such as trauma-focused cognitive-behavioral therapy (TF-CBT), can be particularly beneficial in helping these women manage their post-traumatic symptoms and reduce their substance use.

**Disclosure of Interest:**

None Declared

